# A three‐gene signature from protein–protein interaction network of *LOXL2*‐ and actin‐related proteins for esophageal squamous cell carcinoma prognosis

**DOI:** 10.1002/cam4.1096

**Published:** 2017-05-29

**Authors:** Xiu‐hui Zhan, Ji‐wei Jiao, Hai‐feng Zhang, Chun‐quan Li, Jian‐mei Zhao, Lian‐di Liao, Jian‐yi Wu, Bing‐li Wu, Zhi‐yong Wu, Shao‐hong Wang, Ze‐peng Du, Jin‐hui Shen, Hai‐ying Zou, Gera Neufeld, Li‐yan Xu, En‐min Li

**Affiliations:** ^1^ Department of Biochemistry and Molecular Biology Shantou University Medical College Shantou Guangdong China; ^2^ The Key Laboratory of Molecular Biology for High Cancer Incidence Coastal Chaoshan Area Shantou University Medical College Shantou Guangdong China; ^3^ College of Medical Informatics Daqing Campus Harbin Medical University Daqing Heilongjiang China; ^4^ Institute of Oncologic Pathology Shantou University Medical College Shantou Guangdong China; ^5^ Department of Tumor Surgery Shantou Central Hospital Affiliated Shantou Hospital of Sun Yat‐sen University Shantou Guangdong China; ^6^ Department of Pathology Shantou Central Hospital Affiliated Shantou Hospital of Sun Yat‐sen University Shantou Guangdong China; ^7^ Cancer Research and Vascular Biology Center The Bruce Rappaport Faculty of Medicine Technion Israel Institute of Technology Haifa Israel

**Keywords:** Actin‐related proteins, esophageal squamous cell carcinoma, lysyl oxidase‐like 2, prognosis, protein–protein interaction network, three‐gene signature

## Abstract

Current staging is inadequate for predicting clinical outcome of esophageal squamous cell carcinoma (ESCC). Aberrant expression of *LOXL2* and actin‐related proteins plays important roles in ESCC. Here, we aimed to develop a novel molecular signature that exceeds the power of the current staging system in predicting ESCC prognosis. We found that *LOXL2* colocalized with filamentous actin in ESCC cells, and gene set enrichment analysis (GSEA) showed that *LOXL2* is related to the actin cytoskeleton. An ESCC‐specific protein–protein interaction (PPI) network involving *LOXL2* and actin‐related proteins was generated based on genome‐wide RNA‐seq in 15 paired ESCC samples, and the prognostic significance of 14 core genes was analyzed. Using risk score calculation, a three‐gene signature comprising *LOXL2*,*CDH1,* and *FN1* was derived from transcriptome data of patients with ESCC. The high‐risk three‐gene signature strongly correlated with poor prognosis in a training cohort of 60 patients (*P *=* *0.003). In mRNA and protein levels, the prognostic values of this signature were further validated in 243 patients from a testing cohort (*P *=* *0.001) and two validation cohorts (*P *=* *0.021, *P *=* *0.007). Furthermore, Cox regression analysis revealed that the signature was an independent prognostic factor. Compared with using the signature or TNM stage alone, the combined model significantly enhanced the accuracy in evaluating ESCC prognosis. In conclusion, our data reveal that the tumor‐promoting role of *LOXL2* in ESCC is mediated by perturbing the architecture of actin cytoskeleton through its PPIs. We generated a novel three‐gene signature (PPI interfaces) that robustly predicts poor clinical outcome in ESCC patients.

## Introduction

Esophageal cancer is the sixth most common cause of death from cancer worldwide, and consists of two primary cancer types, esophageal adenocarcinoma and esophageal squamous cell carcinoma (ESCC). Cancer of the esophagus is a highly aggressive malignancy with very poor survival, and where the overall ratio of mortality to incidence is as high as 0.88 1. The current staging system is inadequate for predicting outcome of treatment, and the addition of cancer biology will make great sense to ESCC therapeutic discovery 2.

Lysyl oxidase‐like 2 (*LOXL2*), a member of lysyl oxidase family, is involved in extracellular matrix (ECM) remodeling, transcription regulation, tumor progression, and metastasis in various types of carcinomas 3, 4, 5, 6. We previously demonstrated that *LOXL2*, with reduced nuclear and ectopic cytoplasmic expression, plays a pivotal role in lymph node metastasis and poor prognosis in ESCC 7. There is also current evidence that intracellular, but not secreted *LOXL2*, promotes migration and invasion of clear cell renal cell carcinoma 8. Thus, it is worth considering that the intracellular (cytoplasmic) distribution of *LOXL2* in ESCC may have a novel and complex biological function independent of its established secreted role.

On the other hand, a large amount of protein–protein interactions (PPIs) participates in driving tumorigenesis through the regulation of oncogenic networks 9. Some cancer‐associated PPI network (PPIN) maps have been established by large‐scale genomic experiments 10. Actins are highly conserved proteins that are involved in maintenance of the cytoskeleton, in which Actin beta (encoded by *ACTB*) and Actin gamma 1 (encoded by *ACTG1*) are found as the two nonmuscle cytoskeletal actin in vertebrates 11. Moreover, aberrant expression of actin‐related proteins plays an important role in tumor progression as the actin cytoskeletal architecture impacts many cancer‐related cellular processes 12, 13. Technological advances in the field of bioinformatics, including microarray analysis of gene expression, now enable the identification of molecules and important prognostic PPIs in clinical settings.

In our current study, we investigated the prognostic value of a signature, based on biological experiments and PPIN, among different clinical cohorts in ESCC. We found that *LOXL2* is related to actin cytoskeleton function in ESCC cells. Subsequently, we examined the prognostic significance of 14 candidate proteins from *LOXL2*‐ and actin‐related proteins within ESCC‐specific PPIN. Moreover, a developed risk score calculating strategy was adopted and a three‐gene signature was derived to accurately predict overall survival in ESCC.

## Material and Methods

### Cell lines and cell culture

Human ESCC cell lines KYSE150 14 and SHEEC 15 were used in the study. KYSE150 cells were cultured in RPMI‐1640 medium with 10% fetal bovine serum, whereas SHEEC cells were maintained in high‐glucose DMEM medium with 4.0 mmol/L l‐Glutamine and Ham's Nutrient Mixture F12 with 10% newborn bovine serum.

### Patients and tissue specimens

Human ESCC tumors and adjacent nontumorous esophageal epithelial tissues were collected directly after surgical resection between January 2000 and January 2013, at Shantou Central Hospital (Sun Yat‐sen University, Shantou, China). Fifteen paired tissues between 2012 and 2013 were used for genome‐wide transcriptome sequencing (RNA‐seq, SPA accession number: SRP064894, Shanghai Biotechnology Corporation, Shanghai, China) 16. We validated the three‐gene signature using a validation tissue microarray cohort of 124 randomly selected patients with clear pathologic diagnosis and follow‐up information (Table S1). All tumors were confirmed as ESCC by pathologists in the Clinical Pathology Department of the hospital, and the tissue specimens were classified according to the tumor‐node metastasis (TNM) classification of the International Union against Cancer. Written informed consent was obtained from all patients or their appropriate surrogates. The study was approved by the ethical committee of Shantou Central Hospital and Shantou University Medical College.

### Gene expression data

Published gene expression datasets, comprising 316 patients of ESCC in total, were from different centers and platforms in the study. RNA‐seq data and clinical information of 84 patients with ESCC were downloaded from The Cancer Genome Atlas (TCGA) database 17. The gene microarray expression profiles and corresponding clinical information of patients with ESCC were obtained from the publicly available Gene Expression Omnibus (GEO) database, including 53 patients from GSE23400 (Affymetrix HG‐U133A array) 18, 119 patients from GSE53624 (Agilent human lncRNA+mRNA array V.2.0), and 60 patients from GSE53622 (Agilent human lncRNA+mRNA array V.2.0) 19. For prognostic gene signature analysis, the 119 specimens in GSE53624 were randomly divided into two groups by the stratified randomization function from SAS (r) Proprietary Software 9.2 (Table S2). One group was used as a training cohort (*n* = 60) to develop a multigene signature for ESCC specimens, whereas the other one was a testing cohort (*n* = 59). GSE53622 (*n* = 60) was provided as a validation cohort for assessing the prediction performance of the signature. The analyses were done blinded from clinical and biologic data and sample identification.

### Immunofluorescence

KYSE150 and SHEEC cells were cultured for 24–48 h. When cells reached 80–90% confluence, they were fixed in 4% paraformaldehyde in phosphate‐buffered saline (PBS) for 10 min, rinsed three times with PBS, and permeabilized in 0.1% Triton X‐100 for 10 min or less. Cells were washed with PBS and blocked in blocking serum (Life Technology, Shanghai, China) for 60 min at room temperature. Cells were stained overnight with anti‐*LOXL2* antibody (1:100; Novus, Littleton, CO), anti‐*LOXL2* C‐terminal antibody (1:100; Abcam, Cambridge, UK), or anti‐FlagM2 (1:200; Sigma‐Aldrich Guangzhou, China) in blocking buffer, and then probed with Alexa Fluor 488‐conjugated Affinipure donkey antirabbit secondary antibody (1:200; Jackson ImmunoResearch, West Grove, PA) and Actin‐stain^™^ 555 phalloidin (1:200; Cytoskeleton, Denver, CO), followed by counterstaining with 0.1 *μ*g/mL 4′,6‐diamidino‐2‐phenylindole (Sigma Aldrich, Guangzhou, China). Confocal imaging was performed on a laser‐scanning confocal microscope (LSM 880; Carl Zeiss, Oberkochen, Germany).

### Bioinformatics analysis

Genes involved in regulation of the actin cytoskeleton were obtained from KEGG data (Release 76.0, KEGG database/map04810, gene numbers = 181). Gene set enrichment analysis (GSEA2.2.0, Cambridge, MA), a powerful analytical method, was performed using Pearson's correlation to determine the degree of linear relationship between *LOXL2* and actin cytoskeleton genes.

A PPI human reference set of 224,522 PPIs between 18,130 proteins was obtained from the Biological General Repository for Interaction Datasets (BioGRID, Release 3.4), and was mapped into Cytoscape software (Version 3.2.1, http://www.cytoscape.org) as a visualizing parent PPI network. *LOXL2* PPIs from variable resources and PPIs of *ACTB* or *ACTG1* were extracted from the parent PPIN to generate a *LOXL2*‐*ACTB*/*ACTG1* PPIN. The network then created a *LOXL2*‐*ACTB*/*ACTG1* PPIN in ESCC with a filtering method according to reads per kilobase of exon per million mapped reads (RPKM) of the RNA‐seq of ESCC specimens. When the RPKM of one gene in 15 tumor samples is higher than 10, the gene will be regarded as an effective detecting candidate for the following analysis. Self‐loops and duplicated edges of proteins in the networks were removed. Gene Ontology (GO) and KEGG pathway assignments were performed using Gene Functional Classification Tool from the DAVID Bioinformatics Resources database (Release 6.7). The GO terms in the Biological Process (GOTERM‐BP‐FAT) and KEGG pathways with *P* < 0.05 and enrichment score > 2 were considered as significantly enriched function annotations.

### Tissue Microarrays and Immunohistochemistry

Tissue microarray (TMA) construction of esophageal carcinoma tissue has been described in our previous study 20. The SuperPicTure Polymer Detection Kit and the Liquid DAB Substrate Kit (Zymed/Invitrogen, Carlsbad, CA) were used to conduct immunohistochemistry according to the manufacturer's instructions. Primary antibodies used for immunohistochemistry staining in this study are listed below: anti‐*LOXL2* antibody (1:100; Abnova, Taipei, China), anti‐ Fibronectin antibody (1:200; Santa Cruz Biotechnology, Dallas, Texas), and anti‐E‐cadherin (1:100; BD Biosciences, Franklin Lakes, NJ). The TMAs were intelligently scanned by a multimodal and high‐throughput imaging system, Vectra 2.0.8 (PerkinElmer Inc., Hopkinton, MA). Following the manufacturer's protocols, the expressions of three proteins were quantified using the Nuance v3.0 incorporated with proprietary image analysis software, inForm v1.2 (PerkinElmer Inc., Hopkinton, MA). First, we built a tissue segmentation algorithm to separate cancer from its surrounding stroma in multispectral image of stained TMAs sections. Then, the tumor cells were circled individually and the amount of staining intensity was measured and digitized. Through a series of analysis above, the sum scores of each protein expression in the three‐gene signature of individual tissue specimens were obtained (range from 0 to 300) and subjected to statistical analysis.

### Calculation of risk scores

The freely available gene expression data of ESCC specimens in GSE53624 and GSE53622 were already log2 scale transformed 19. High expression or low expression of a given gene was defined on the basis of the level of mRNA log2 expressions by the automatic optimum selection method in X‐tile software (Release 3.6.1), and the same was applied to immunohistochemistry scores of the protein. In all cohorts in the study, each gene of each patient was assigned a numerical value in which high expression equaled 1 and low expression equaled 0. To estimate overall survival of a signature formed by *LOXL2*,* CDH1,* and *FN1* genes, we adopted a developed strategy using a univariate Cox proportional hazards regression analysis coefficient for the three genes 21. A given patient's prognosis risk score was then calculated as the sum of each gene's score, which was derived by multiplying the gene expression level by its corresponding regression coefficient (prognostic risk score = ΣCox coefficient of gene Gi × expression value of gene Gi). Finally, the patients were separated into two groups (high‐risk signature and low‐risk signature) using the 50th percentile cut‐off of the risk score of the three‐gene signature as a threshold. To reduce the influence of heterogeneity among different patients or cohorts, the risk scores and the cut‐off value derived from the training cohort were reestimated and then applied to the other three cohorts.

### Statistical analysis

Unless otherwise stated, statistical analyses were performed with SPSS 22.0 software (IBM, Chicago, IL) or GraphPad Prism 5 software (La Jolla, CA). The differences between groups were analyzed by the Student *t* test or paired‐samples *t* test. The chi‐squared test or Fisher's exact test was used to assess the correlations between category variables. Overall survival (OS) was estimated by the Kaplan–Meier method. Multivariate Cox proportional hazards regression (HR) analysis was applied to evaluate independent prognostic factors associated with overall survival, and the three‐gene signature, age, gender, and tumor stage, was used as covariates. To estimate the sensitivity and specificity of the signature and area under the curve (AUC) value, receiver operating characteristic (ROC) analysis was adopted in three cohorts. The differences between the AUC of the three‐gene signature, TNM stage, and a combination of both were detected by MedCalc software (Vision 15.8, Ostend, Belgium). A *P* < 0.05 was considered to indicate statistical significance, and all statistical tests were two‐sided.

## Results

### *LOXL2* is related to the actin cytoskeleton in ESCC

**Figure 1:**
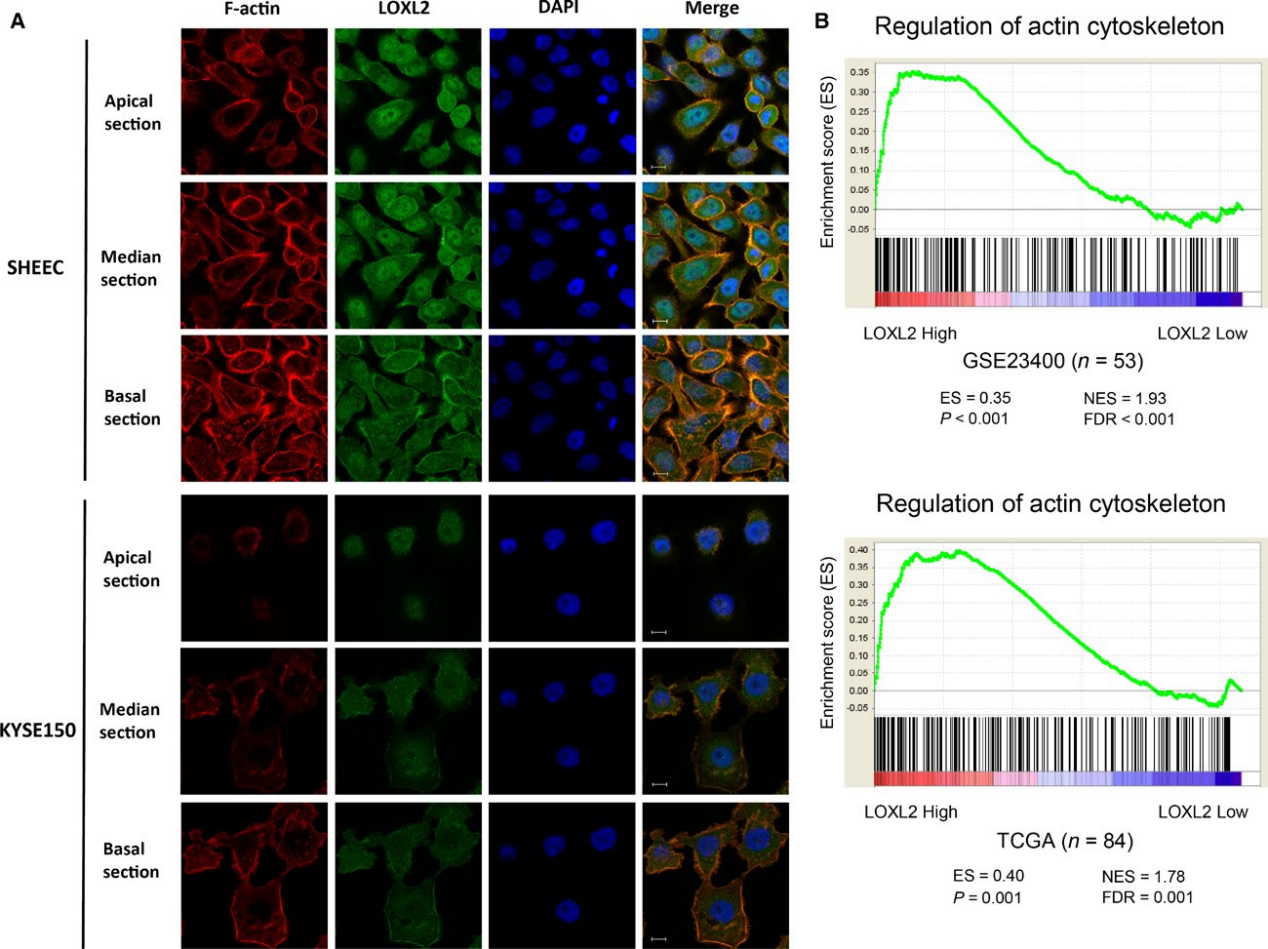
Association of *LOXL2* and actin cytoskeleton in ESCC. (A) Colocalization of *LOXL2* and F‐actin in confocal microscopy of ESCC cell lines with apical, median, and basal focal xy section images. Scale bar = 10 *μ*m. ESCC, esophageal squamous cell carcinoma. (B) GSEA plots showing that *LOXL2* expression positively correlates with regulation of actin cytoskeleton genes (KEGG database/map04810, gene numbers = 181) in a published ESCC patient gene expression profile (NCBI/GEO/GSE23400, *n* = 53) and an RNA‐seq profile (TCGA esophageal carcinoma, *n* = 84). TCGA, The Cancer Genome Atlas, USA; ES, enrichment score; FDR, false discovery rate; NES, normalized enrichment score.

To explore the subcellular location of *LOXL2* in ESCC, we studied the human ESCC cell lines KYSE150 and SHEEC. *LOXL2* in SHEEC cells was mainly localized at the median and basal cytoplasm and nucleus, but weak staining was also observed at the apical region (Fig. 1A upper panel). Similar staining of *LOXL2* was detected in KYSE150 cells, except for a strong signal in the nucleus (Fig. 1A lower panel). To our great surprise, *LOXL2* and filamentous actin (F‐actin) were colocalized in ESCC cells (Fig. 1A and S1). Although F‐actin staining was abundant throughout the cells, colocalization of *LOXL2* and F‐actin was primarily restricted to the cytoplasm near the cell membrane and a few cell–cell contacts. The gene sets enrichment analysis (GSEA) plots indicated that *LOXL2* expression was positively correlated with regulation of actin cytoskeleton genes in patients with ESCC (Fig. 1B). Altogether, these observations point to a new relationship in which *LOXL2* is related to the actin cytoskeleton in ESCC.

### Development and analyses of a *LOXL2*‐ACTB/ACTG1 PPI network in ESCC

**Figure 2:**
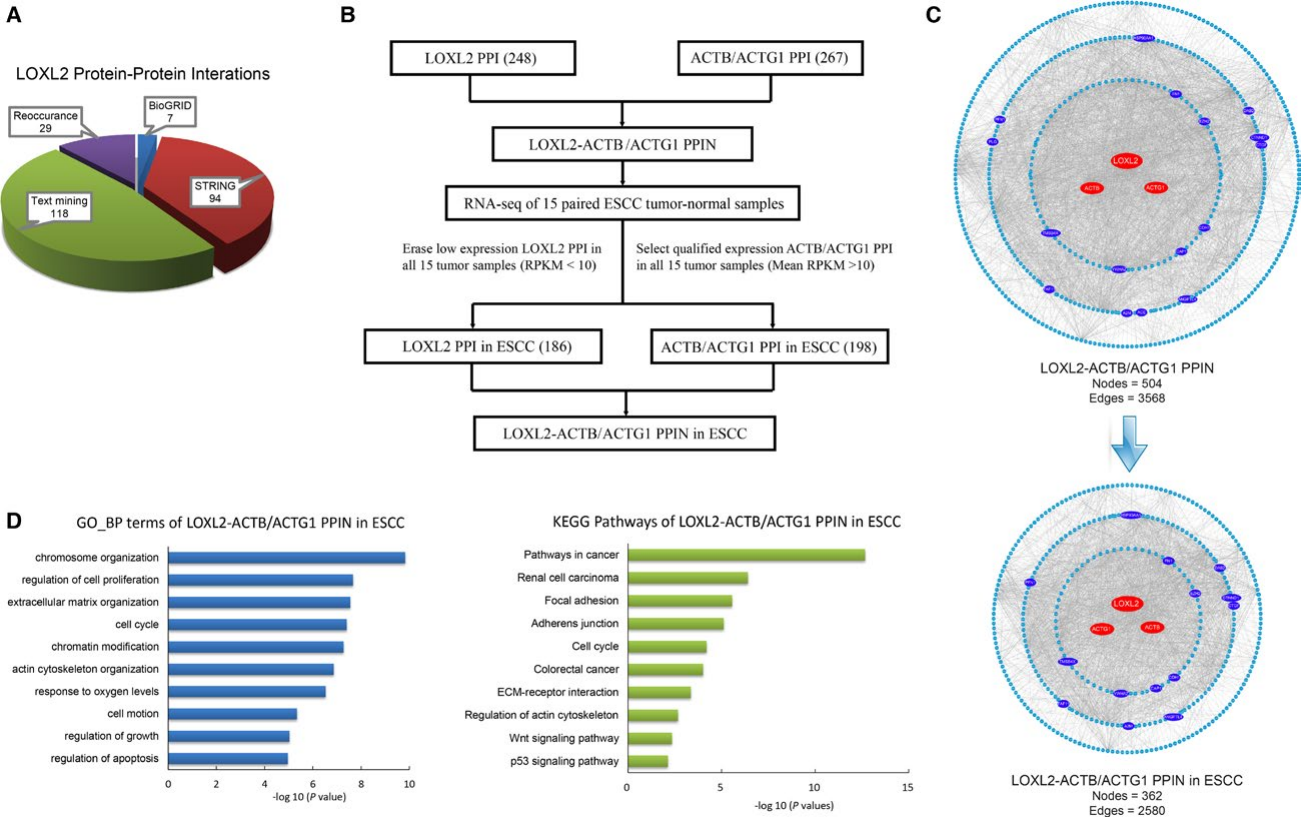
Development and analyses of the *LOXL2*‐ACTB/ACTG1 protein–protein interaction network in ESCC. (A) Different sources of certain and potential *LOXL2* protein–protein interactors. (B) Method for developing the *LOXL2*‐*ACTB*/*ACTG1* protein–protein interaction network in ESCC. The node numbers in brackets mean gene numbers. PPI, protein–protein interactor; RPKM, reads per kilobase of exon per million mapped reads. (C) PPI subnetwork generation by mapping *LOXL2* PPIs and *ACTB*/*ACTG1* PPIs to the BioGRID parental PPI network. *LOXL2*‐*ACTB*/*ACTG1* PPIN in ESCC (lower panel) was extracted from the total *LOXL2*‐*ACTB*/*ACTG1* PPIN (upper panel) with the method in (B). Different colors of nodes indicate different types of proteins. Red nodes represent central proteins in the network. Dark blue nodes represent 14 core interacting proteins. Light blue nodes represent common proteins in the network. PPIN, protein–protein interaction network. (D) Significant patterns for biological process (BP) GO terms and KEGG pathways of the *LOXL2*‐*ACTB*/*ACTG1* PPIN in ESCC.

Based on the work above, we assumed that the relevance of *LOXL2* and actin cytoskeleton in ESCC is through a number of their interacting proteins. We gathered certain and potential *LOXL2* protein–protein interactors (PPIs) from variable protein databases and published reports (Fig. 2A). All these and acknowledged human PPI data from the BioGRID database were combined to provide the original data for subsequent analyses. A *LOXL2*‐*ACTB*/*ACTG1* PPIN, from all human tissues, containing 504 nodes and 3568 edges was generated by mapping total *LOXL2*,* ACTB,* and *ACTG1* PPIs to the parental PPI network (Fig. 2C left panel). To create an ESCC‐specific subnetwork, we examined 21,157 gene expressions in RNA‐seq of 15 patients with ESCC. The low‐expressional ones out of 504 proteins in ESCC tissues were excluded through an RPKM‐filtering method, and the remaining 2580 PPIs between 362 proteins were simultaneously selected for the following survey (Fig. 2B, C right panel).

We found that the genes in the ESCC‐specific network were intimately connected to regulation and reorganization of the cytoskeleton, as well as multiple typical carcinoma‐related cell behaviors, such as cell adhesion, cell invasion, and cell migration (Fig. 2D and Table S3). To better know the network composition of the ESCC‐specific network, we grouped the proteins in three subcategories upon the network topological properties: central proteins consisting of *LOXL2*,* ACTB,* and *ACTG1*, core proteins comprising 14 interacting proteins, and other 345 common proteins (Fig. 2C).

### Derivation of a three‐gene signature predictive of survival in ESCC

The 14 *LOXL2*‐*ACTB*/*ACTG1* PPIN core proteins are listed as below: *A2M*,* ANGPLT4*,* CAP1*,* CDH1*,* CTGF*,* CTNND1*,* DAB2*,* EZH2*,* FN1*,* HSP90AA1*,* PFN1*,* TAF11*,* TMSB4X,* and *YWHAZ*. Due to its absence in microarray expression profiles in our study (GSE53624 and GSE53622), the core protein *PFN1* was left out at first. Central gene *LOXL2* and the other 13 core genes were considered as potential ESCC prognostic biomarkers and were further studied.

**Figure 3:**
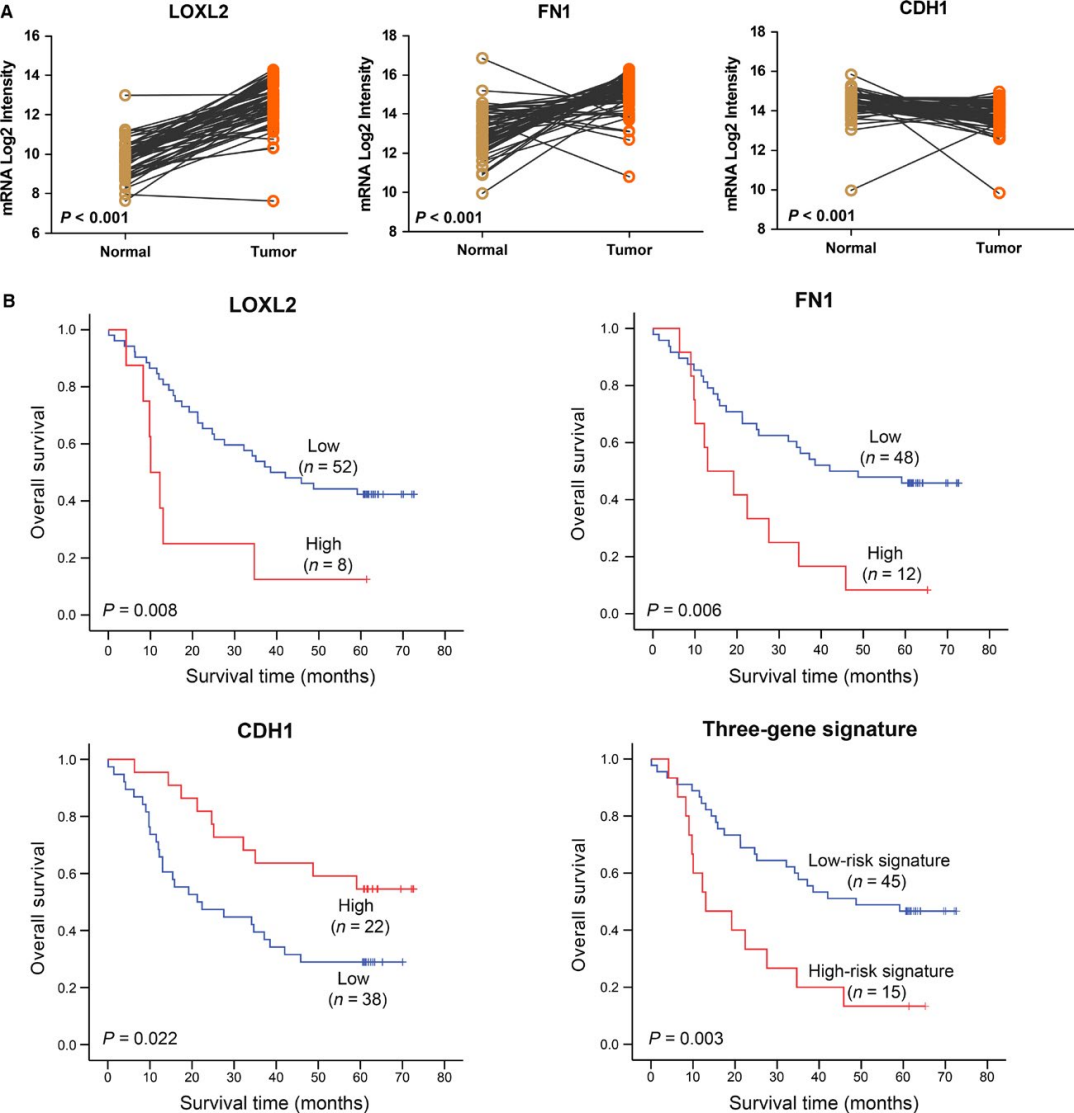
Derivation of a three‐gene signature in a training cohort of ESCC patients. (A) *LOXL2*,*CDH1,* and *FN1* are differentially expressed between 60 ESCC tumors and paired normal specimens in the training cohort (statistical significance assessed by the paired‐samples *t* test). (B) Kaplan–Meier curves of three genes (*LOXL2*,*CDH1,* and *FN1*) separately and the three‐gene signature for overall survival in the training cohort. Three‐gene signature prediction of high risk predicts poor survival in patients with ESCC.

For prognostic gene signature analysis, we obtained microarray data from 119 patients with ESCC (GEO accession number: GSE53624) and randomly assigned it to either the training cohort or the testing cohort. Among the 60 cases in the training cohort, potentially crucial genes, excluding *CTGF*,* DAB2,* and *TMSB4X*, were significantly differentially expressed between ESCC tumors and paired normal specimens (Fig. 3A and Fig. S2). Five from the differentially expressed genes were upregulated in tumor tissues, whereas the others were downregulated in contrast. Moreover, high‐level expression of five genes was found to be significantly associated (*P *<* *0.05 by the log‐rank test) with outcome of patients with ESCC in the training cohort: four genes (*FN1*,* LOXL2*,* TAF11*, and *TMSB4X*) were associated with shorter OS and high‐level expression of one gene, *CDH1*, was associated with a better clinical outcome (Fig. 3B and S3).

Summarizing the analytic results in the training cohort, *LOXL2*,* CDH1*,* FN1,* and *TAF11* were in accordance with the inclusive criteria. On account of the biological functions of these genes, we ultimately selected *LOXL2* (lysyl oxidase‐like 2), *CDH1* (E‐cadherin 1, type 1), and *FN1* (fibronectin 1) to integrate a three‐gene signature in ESCC. Afterward, the three‐gene signature in the patients among the training cohort was evaluated with a risk score algorithm provided by univariate Cox regression. The Cox coefficients for *LOXL2*,* CDH1,* and *FN1* were 1.089, 0.980, and −0.827, respectively. The 50th percentile of the prognostic risk scores was 0 (range from −0.827 to 2.069), therefore, all individuals were classified into two groups: high‐risk signature (risk score > 0) and low‐risk signature (risk score ≤ 0). Patients with a low‐risk gene signature (*n* = 45) had a longer median overall survival than patients with a high‐risk gene signature (*n* = 15) (42.0 months vs. 23.3 months, *P *=* *0.003) (Fig. 3B). Multivariate Cox proportional hazard regression analyses revealed that mortality risk assessed by the three‐gene signature was, along with tumor stage, an independent predictor of survival (*P *<* *0.001, HR = 4.48, 95% CI = 2.03–9.90) among ESCC patients in the training cohort (Table 1). In conclusion, high risk, predicted by the three‐gene signature, predicts poor survival in ESCC patients.

**Table 1:** Hazard ratios for death from ESCC among four cohorts, according to multivariate Cox regression analysis^a^

Variable	Hazard Ratio (95%CI)	*P*‐value
Training cohort		
High‐risk three‐gene signature	4.48 (2.03‐9.90)	< 0.001
Tumor stage III	2.76 (1.32‐5.81)	0.007
Testing cohort		
High‐risk three‐gene signature	2.87 (1.47‐5.61)	0.002
Validation cohort		
High‐risk three‐gene signature	2.21 (1.07‐4.58)	0.032
Older age	2.12 (1.03‐4.36)	0.042
Validation tissue microarray cohort		
High‐risk three‐gene signature	1.88 (1.13‐3.14)	0.016
Tumor stage III	2.75 (1.62‐4.68)	< 0.001

a Variables were selected with a stepwise selection method. CI denotes confidence interval.

### Validation of the three‐gene signature and survival

**Figure 4:**
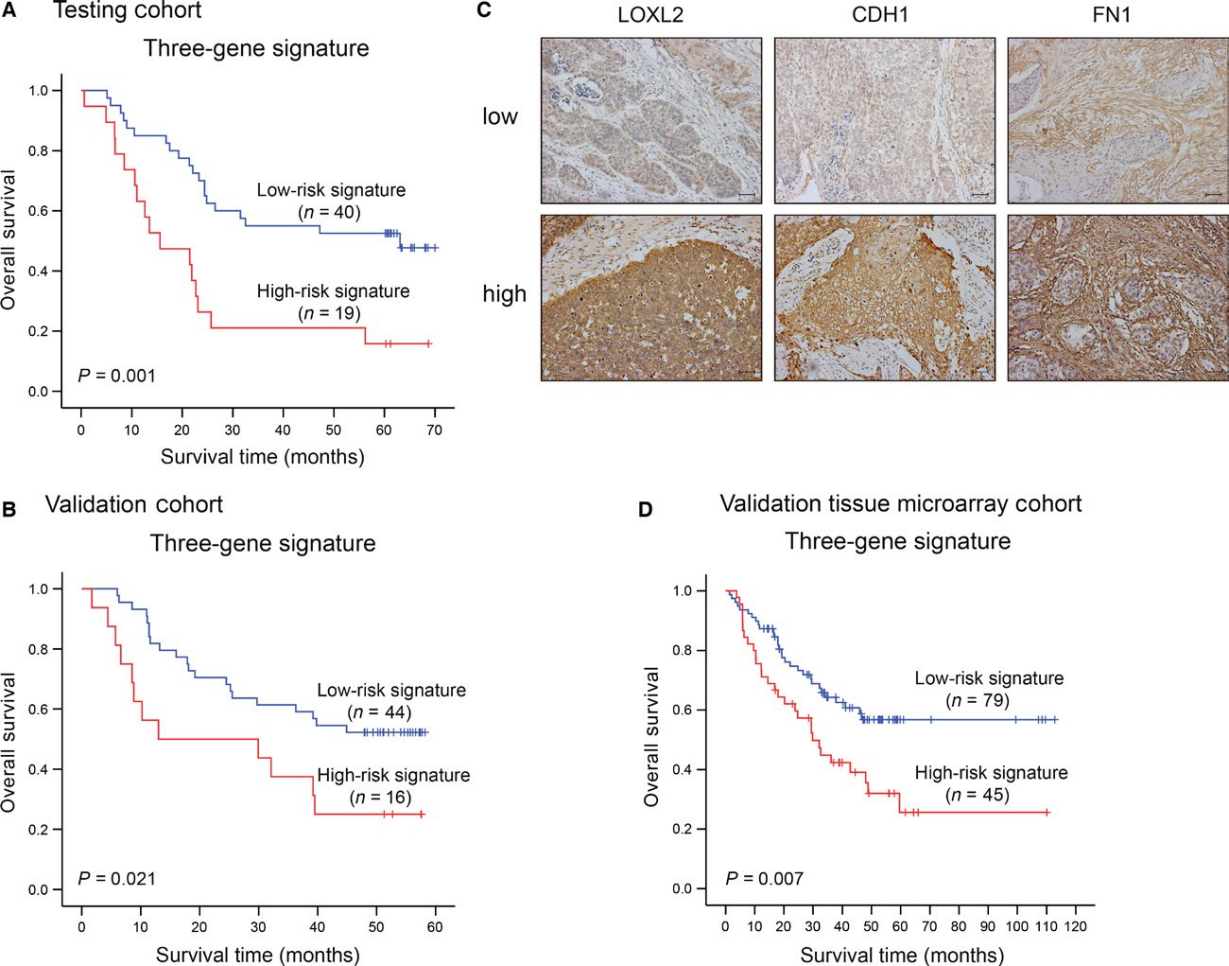
Validation of the three‐gene signature. Kaplan–Meier estimates of overall survival of ESCC patients according to the three‐gene signature in the testing cohort (A) and the validation cohort (B). (C) Representative low/high expression of *LOXL2*,*CDH1,* and *FN1* by immunochemistry study in tissue microarrays. The bar indicates 50 *μ*m. (*D*) Kaplan–Meier curves of the three‐gene signature for overall survival in the validation tissue microarray cohort.

To test the three‐gene signature for its prognostic value, we applied it to the microarray data of the testing cohort. The same risk score algorithm and criteria, as which were derived from the training cohort, classified 19 and 40 ESCC patients of the testing cohort into the high‐risk and low‐risk groups, respectively. The high‐risk gene signature was associated with a median OS of 23.8 months, whereas the low‐risk signature was associated with a median OS of 43.0 months (*P *=* *0.001 by the log‐rank test) (Fig. 4A and S4). The three‐gene signature was then validated in another cohort comprising 60 patients with ESCC (GEO accession number: GSE53622). Similar to the above, patients in the low‐risk group had significantly longer survival time than those in the high‐risk group (median survival months: 37.7 vs. 26.2, *P *=* *0.021) (Fig. 4B and S5A). The high‐risk three‐gene signature remained significantly associated with shorter overall survival in the testing cohort (*P *=* *0.002, HR = 2.87, 95% CI = 1.47–5.61) and the validation cohort (*P *=* *0.032, HR = 2.21, 95% CI = 1.07–4.58) (Table 1).

Not only in the mRNA level, but the three‐gene signature was further validated in the protein level with a tissue microarray cohort of 124 patients with ESCC. Each protein displayed two immunostaining phenotypes; that is, low staining and high diffuse staining (Fig. 4C). The OS in the high‐risk signature group was significantly shorter than that in the low‐risk signature group (median survival months: 46.5 vs. 73.6, *P *=* *0.007) (Fig. 4D and Fig. S5B). Most importantly, the high‐risk group predicted by three‐gene signature also represented a higher mortality risk than the low‐risk group (*P *=* *0.016, HR = 1.88, 95% CI = 1.13‐3.14) (Table 1). Therefore, the three‐gene signature is a robust high‐risk factor for ESCC prognosis.

### Combination of the three‐gene signature and TNM stage among the four cohorts

**Figure 5:**
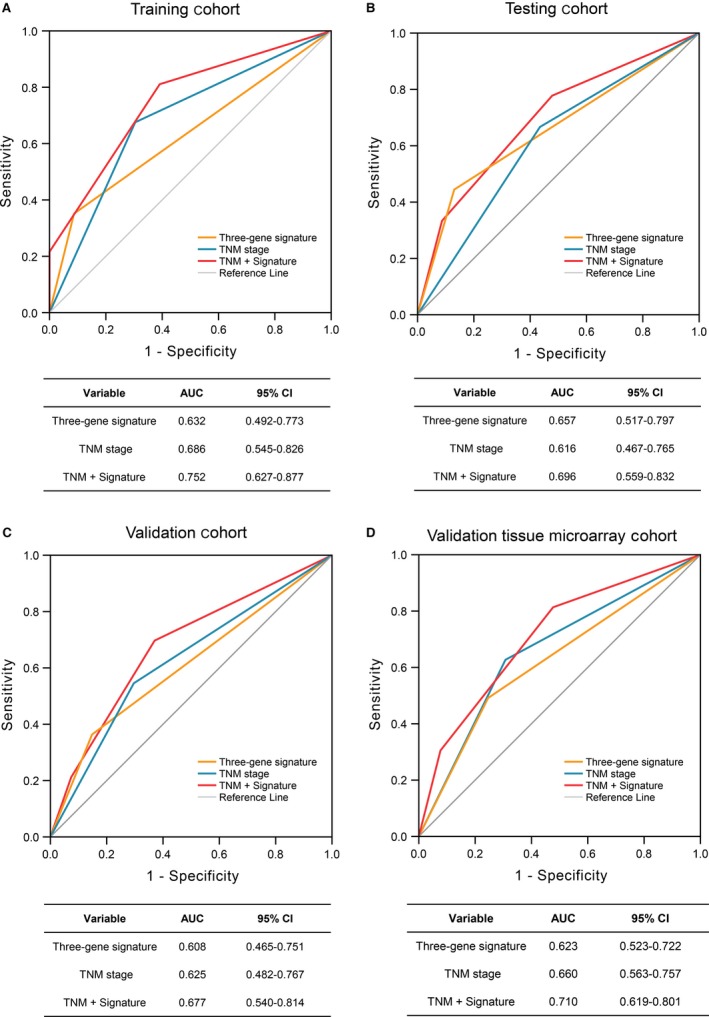
The predictive ability of the three‐gene signature. The four receiver operating characteristics (ROC) curves in the training cohort (A), the testing cohort (B), the validation cohort (C), and the validation tissue microarray cohort (D). Comparison of sensitivity and specificity for survival prediction by the three‐gene signature, TNM stage, and combination of the two factors. AUC shows the area under the ROC. CI, confidence interval; TNM, pathologic tumor, node, and metastasis.

In current clinical analysis, TNM stage classification is deemed to be the optimal prognostic indicator for cancer. As demonstrated in Table S4 and S5, there was no statistically evident relationship between expression level of the three‐gene signature and any ESCC clinical characteristic in not only the testing cohort but also in the three validation cohorts. A prognostic model combining TNM stage and the three‐gene signature was conducted and receiver operating characteristic (ROC) analysis was performed. The combined model had a higher area under curve (AUC) than both the signature and the TNM stage alone in the four cohorts (Fig. 5). In applying this criteria to ESCC patients, we found that the predictive ability of the combined model was significantly better than the signature or the TNM stage alone. As an example, the predictive ability between the combined model and TNM stage alone was 0.696 versus 0.616 (*P *<* *0.05, Fig. 5B).

## Discussion

China has high rates of cancer mortality, of which esophageal cancer is the fourth most prevalent cancer, with a 5‐year relative survival below 30% 22, 23. In our previous study, we discovered that *LOXL2* is mainly expressed in the cell cytoplasm in ESCC tissues and cells 7, 24. In this study, we reconfirmed the intracellular location of *LOXL2* protein in ESCC, which is separated from its conventional location in extracellular space 25. We found that *LOXL2* and filamentous actin (F‐actin) colocalized in the cytoplasm near the cell membrane and inside ESCC cells near sites of cell–cell contact. As one of the three main cytoskeletal polymers, F‐actin carries out central functions in cell structure, intracellular organization, and cell movement 26. While cancer cells regulate their cytoskeletal structures, they stimulate cellular motility to metastasize away from the initial niche and migrate into adjacent tissues, and even invade distant tissues 27. As we expected, *LOXL2* expression positively correlates with regulation of the actin cytoskeleton in ESCC patients. Recent studies using loss‐ or gain‐of‐functions approaches also indicate that *LOXL2* regulates actin cytoskeletal reorganization in several cancers. *LOXL2* cross‐links ECM components and increases tissue stiffness, thereby enhancing the reorganization of cytoskeleton in hepatocellular carcinoma cells 28. Whereas, *LOXL2* knockdown markedly reduces stress fiber and focal adhesion formation in human clear cell renal cell carcinoma cells 8.

From another perspective, the number of high‐quality binary PPIs in the literature has grown roughly linearly, and publications about cancer‐related PPIs has also risen rapidly since the 1990s 9, 29. As proteins almost never perform their function alone, analyzing their network‐based protein function can give valuable insight into their roles in a cell 30. Here, we developed an ESCC‐specific PPIN of *LOXL2*‐ and actin‐related proteins through RPKM filtering based on the RNA‐seq data of 15 patients with ESCC. Genes in the ESCC‐specific network are primarily involved in regulation and reorganization of the actin cytoskeleton, as well as common cancer‐related cell behaviors. Therefore, we suggest that the intracellular (cytoplasmic) distribution of *LOXL2* in ESCC probably has a novel biological function, independent of its classical secreted role as extracellular *LOXL2*, whereby *LOXL2* indirectly causes perturbation of actin cytoskeleton through its PPIs in ESCC, and contributes to hyperactivation or loss‐of‐function of some key proteins in carcinogenic pathways to enhance oncogenicity. Nevertheless, further research is needed for the underlying molecular mechanism.

There is no doubt that immunohistochemistry, tissue microarrays, and RT‐PCR are in general use to provide molecular signatures for cancer diagnosis and prognosis. However, the emergence and convergence of high‐throughput techniques and bioinformatics predictions will expand the landscape of molecular signatures in cancer for therapeutic and prognostic discovery 31, 32, 33. In this study, through the sum of experimental and bioinformatics results, we derived a three‐gene signature (involving *LOXL2*,* CDH1,* and *FN1*), using a risk score calculation based on published microarray data of ESCC specimens. The presence of a high‐risk three‐gene signature in the ESCC patients of the training cohort was strongly associated with a decreased overall survival, and the prognostic capacity was validated in both mRNA and protein levels within a testing and two validation cohorts. Furthermore, the prognostic signature compensates for current ESCC staging and improves precision for prognosis of ESCC patients. Meanwhile, the signature more effectively predicted survival when combined with TNM stage. Thus, we believe that the prognostic capacity of the three‐gene signature is amenable to patients with ESCC.

Lysyl oxidase‐like 2 (*LOXL2*), a copper‐dependent amine oxidase known to initiate cross‐linking of collagen and elastin, induces desmoplastic stroma, fibrosis, dedifferentiation, and tumor progression in several solid cancers, including in ESCC 6, 34, 35. E‐cadherin (epithelial cadherin, also known as *CDH1*, E‐cad) is a calcium‐dependent cell–cell adhesion glycoprotein, and its transassociation in homophilic complexes, supported by actin filaments (F‐actin) through catenin, is required for tissue stability 36, 37, 38. *LOXL2* is necessary and sufficient to repress E‐cadherin under hypoxia condition in cancer 39. Loss of E‐cadherin, considered as a hallmark of EMT, contributes to cancer progression in by increasing proliferation, invasion, and metastasis 40, 41. *LOXL2* interacts with *E47* to functionally collaborate in the repression of E‐cadherin promoter, and fibronectin is essential for this *LOXL2*/*E47*‐mediated modulation of early metastasis colonization 42. Fibronectin 1 (*FN1*) mediates a wide variety of cellular interactions with the ECM, and is involved in cell adhesion, migration processes, and metastasis 43, 44. Moreover, it is reported that altered expression of fibronectin 1 exists in several squamous cell carcinomas, including ESCC, head and neck squamous cell carcinoma, and oral squamous cell carcinoma 45, 46, 47. The derivation of these three genes predictive of clinical outcome in patients with ESCC may reveal PPI anticancer targets for the development of therapy for esophageal cancer. Recently, a *LOXL2*‐specific monoclonal antibody (AB0023/4) was shown to be effective against primary tumor growth and reduce metastatic burden in mouse models 48. Similarly, AB0024 synthesized by Gilead Sciences has begun to be used in clinical trials in humans with advanced solid tumors (ClinicalTrials.gov Identifier: NCT01323933). Since PPI interfaces as an anticancer strategy has become a reality 49, the three‐gene signature, acting as a cancer‐enabling PPI interface, may become a new promising therapeutic target in ESCC.

In conclusion, this is a pioneering study of a molecular signature for cancer prognosis based on both biological experiments and bioinformatics data. For the first time, our data reveal that the tumor‐promoting role of *LOXL2* in ESCC probably perturbs the architecture of the actin cytoskeleton through its PPIs and induces additional oncogenic effects by hyperactivation or loss‐of‐function of key proteins. The three‐gene signature (PPI interfaces), derived by us, is robustly associated with the clinical outcome in patients with ESCC. Whether the development mode of molecular signatures can be universally used requires a study with a sufficient number of other cancers.

## Conflict of Interest

None declared.

## Supporting information


**Figure S1.** Colocalization of *LOXL2* and F‐actin in confocal microscopy of ESCC cell. *(a)* Co‐localization of *LOXL2* (C‐terminal) and F‐actin in confocal microscopy of SHEEC cells with 2D section images. *(b)* Co‐localization of *LOXL2*‐Flag and F‐actin in confocal microscopy of KYSE150 cells with 2D section images.

**Figure S2.** Analysis of 11 other *LOXL2*‐*ACTB*/*ACTG1* PPIN core genes expression between tumor and paired normal samples in the training cohort.

**Figure S3.** Kaplan–Meier curves and log‐rank tests of 11 other *LOXL2*‐*ACTB*/*ACTG1* PPIN core genes for overall survival in the training cohort.

**Figure S4.** Three genes of the signature in a testing cohort of ESCC patients.

**Figure S5.** Kaplan–Meier curves and log‐rank tests of three genes of the signature for overall survival in the validation cohort (*a*) and the validation tissue microarray cohort (*b*).

**Table S1.** Patient demographics and clinical characteristics for validation tissue microarray cohort in ESCC.

**Table S2.** Patient demographics and clinical characteristics for the training cohort, testing cohort, and validation cohort.

**Table S3.** Gene Ontology (GO) biological process and KEGG pathway analysis for genes of *LOXL2*‐*ACTB*/*ACTG1* PPIN in ESCC.

**Table S4.** Clinical and pathological characteristics of patients with ESCC with high‐ or low‐risk three‐gene signature in the training cohort, testing cohort, and validation cohort.

**Table S5.** Clinicopathological characteristics of ESCC patients with high‐ or low‐risk three‐gene signature in the validation tissue microarray cohort (*n* = 124).
