# Supplementary figures and images for: A three‐gene signature from protein–protein interaction network of LOXL2‐ and actin‐related proteins for esophageal squamous cell carcinoma prognosis
Source: Cancer Med. 2017 May 29;6(7):1707–19. doi: 10.1002/cam4.1096 (PMC5504325; doi:10.1002/cam4.1096)

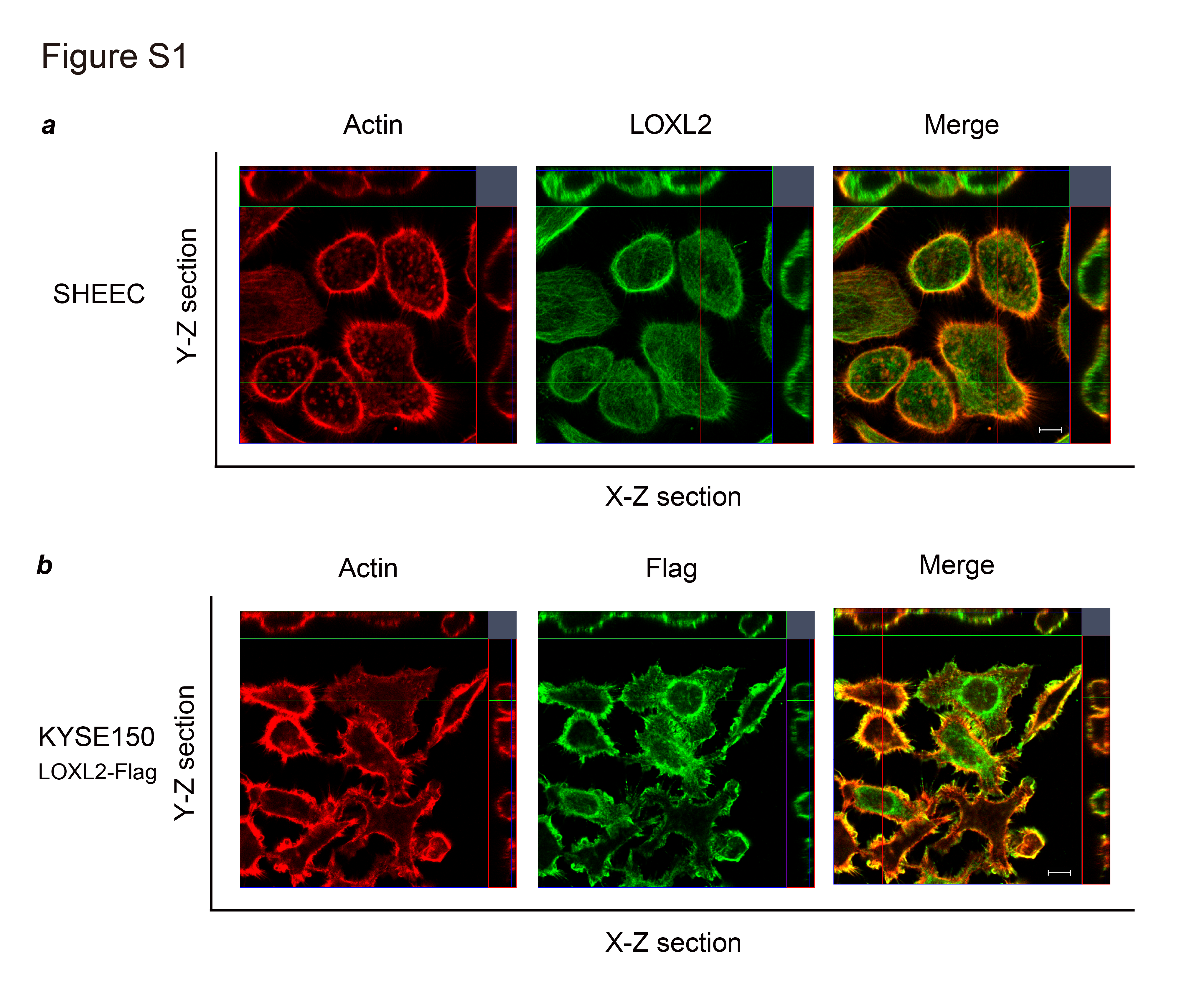

Supplement: Supplementary file 1 — Figure S1. Colocalization of LOXL2 and F‐actin in confocal microscopy of ESCC cell. (a) Co‐localization of LOXL2 (C‐terminal) and F‐actin in confocal microscopy of SHEEC cells with 2D section images. (b) Co‐localization of LOXL2‐Flag and F‐actin in confocal microscopy of KYSE150 cells with 2D section images. [file CAM4-6-1707-s001.tif]

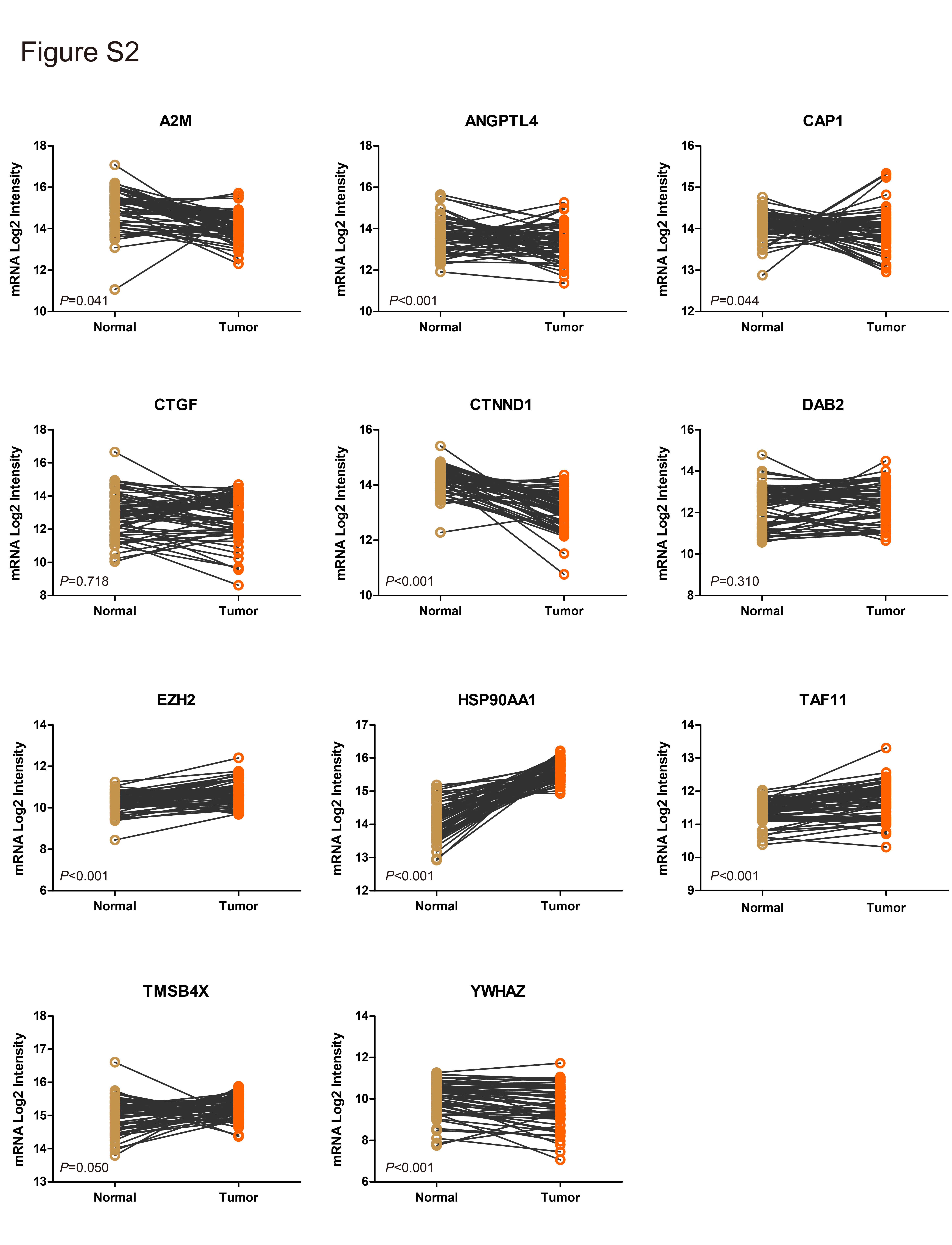

Supplement: Supplementary file 2 — Figure S2. Analysis of 11 other LOXL2‐ACTB/ACTG1 PPIN core genes expression between tumor and paired normal samples in the training cohort. [file CAM4-6-1707-s002.tif]

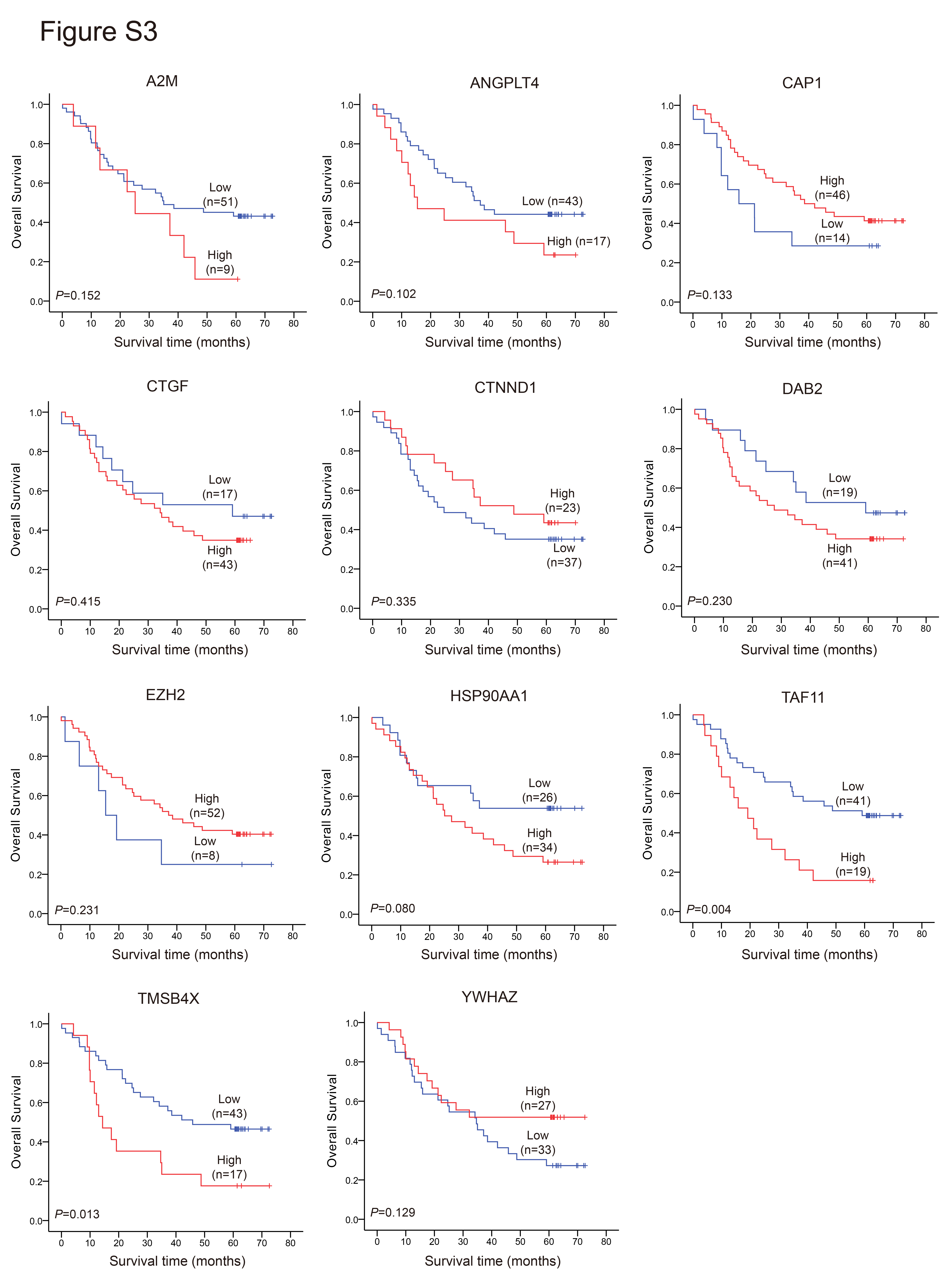

Supplement: Supplementary file 3 — Figure S3. Kaplan–Meier curves and log‐rank tests of 11 other LOXL2‐ACTB/ACTG1 PPIN core genes for overall survival in the training cohort. [file CAM4-6-1707-s003.tif]

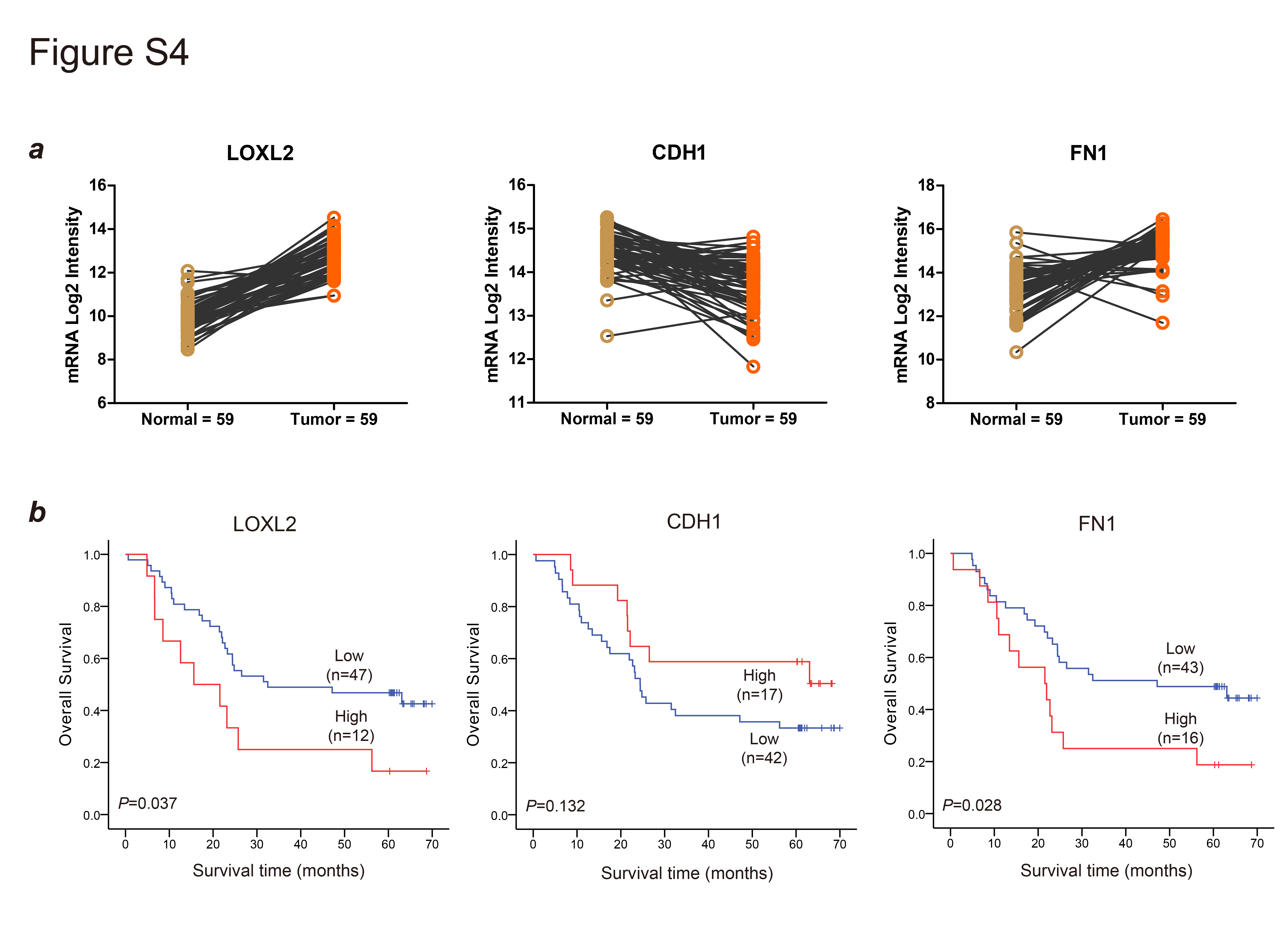

Supplement: Supplementary file 4 — Figure S4. Three genes of the signature in a testing cohort of ESCC patients. [file CAM4-6-1707-s004.tif]

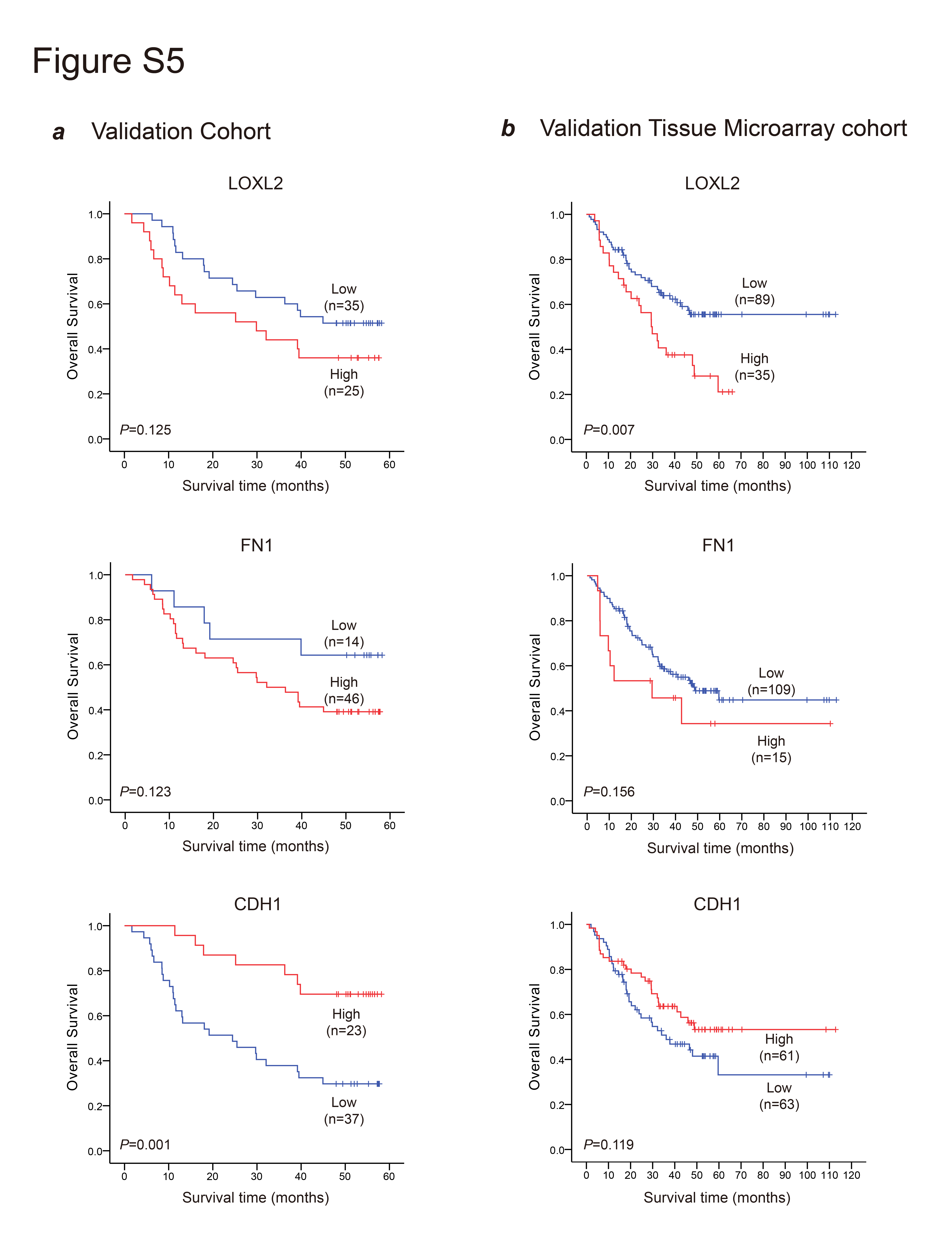

Supplement: Supplementary file 5 — Figure S5. Kaplan–Meier curves and log‐rank tests of three genes of the signature for overall survival in the validation cohort (a) and the validation tissue microarray cohort (b). [file CAM4-6-1707-s005.tif]
